# Lupus-Associated Acute Pancreatitis: A Rare Manifestation of Disease Activity

**DOI:** 10.7759/cureus.114439

**Published:** 2026-08-12

**Authors:** Kenza El Amrani, Hanane Delsa, Nada Faquir, Fatima Belabbes, Imane Ben El Barhdadi

**Affiliations:** 1 Gastroenterology and Hepatology, Cheikh Khalifa International University Hospital, Casablanca, MAR; 2 Faculty of Medicine, Mohammed VI University of Sciences and Health (UM6SS), Casablanca, MAR

**Keywords:** acute pancreatitis, case report, corticosteroids, sledai, systemic lupus erythematosus

## Abstract

Acute pancreatitis affects fewer than 1% of patients with systemic lupus erythematosus (SLE) and usually occurs during active disease. Azathioprine and corticosteroids, both used to treat SLE, can also cause pancreatitis, so the origin of an episode often remains uncertain.

A 41-year-old woman with a two-year history of SLE presented in August 2024 with four days of epigastric pain, diarrhea, and migratory polyarthralgia. She had stopped azathioprine on her own and took hydroxychloroquine and prednisone irregularly. Lipase was 17 times the upper limit of normal, and contrast-enhanced computed tomography showed interstitial oedematous pancreatitis with a CT Severity Index of 2. The Systemic Lupus Erythematosus Disease Activity Index (SLEDAI-2K) score was 20, with low complement, positive anti-dsDNA, and proteinuria at 0.81 g/L. Gallstones, alcohol, hypertriglyceridemia, hypercalcemia, and drug toxicity were excluded. Pain resolved within 48 hours of three pulses of intravenous methylprednisolone, and CRP fell from 49 to 15 mg/L at one month.

Azathioprine had been discontinued before the episode and was subsequently reintroduced without recurrence over 18 months, arguing against drug-induced pancreatitis.

In most reported cases, the suspected agent is continued throughout, so this distinction remains unresolved. The exposure sequence observed here supports attributing the episode to lupus activity and favours intensification of immunosuppression over its withdrawal when SLE is active, and no alternative cause is identified.

## Introduction

Systemic lupus erythematosus (SLE) is a chronic autoimmune disease in which autoantibodies and immune complexes damage multiple organ systems, with a relapsing course alternating flares and remission. It affects mainly women of childbearing age, with a female-to-male ratio of about 9:1 and onset usually between 15 and 45 years. Reported incidence ranges from 1.5 to 8.6 per 100,000 person-years and prevalence from 20 to 366 per 100,000 depending on the region studied. Rates are highest among Black, Hispanic, and Asian populations, who also develop more severe disease [1].

Up to 40% of patients develop gastrointestinal involvement at some point. Lupus enteritis and mesenteric vasculitis are the most frequent forms, while protein-losing enteropathy, hepatitis, and acute pancreatitis are rarer. Separating these from drug toxicity or from causes unrelated to lupus is often difficult [2].

Acute pancreatitis carries mortality ranging from less than 1% in mild interstitial forms to over 30% in severe cases with organ failure [3]. Gallstones, alcohol, and metabolic disturbances, including hypertriglyceridemia and hypercalcemia, account for most cases [4].

In SLE, pancreatitis occurs predominantly during disease flares and carries higher mortality than in the general population [5].

Diagnosis requires excluding common etiologies and accounting for lupus-related medications, particularly azathioprine and corticosteroids, which can themselves trigger pancreatitis [4].

Attributing pancreatitis to lupus activity rather than to azathioprine or corticosteroids is rarely possible with certainty, yet the two lead to opposite decisions: intensifying immunosuppression or withdrawing it. We document this case because the sequence of drug exposure before and after the episode allowed that distinction to be made.

## Case presentation

A 41-year-old woman presented in August 2024 with four days of constant epigastric pain radiating to the back, diarrhea, and migratory polyarthralgia. She had been diagnosed with SLE two years earlier, with initial presentation as a digestive manifestation. Her medical history included lymphopenia, thrombocytopenia, and antiphospholipid syndrome (APS). She had been prescribed hydroxychloroquine, azathioprine, and low-dose prednisone; she had self-discontinued azathioprine and reported inconsistent use of the remaining medications. She denied alcohol use or recent medication changes.

On admission, she was stable and afebrile. Abdominal examination revealed diffuse tenderness. Skin examination showed erythematous macular lesions on the lower limbs, consistent with lupus cutaneous vasculitis.

Epigastric pain radiating to the back in a patient with SLE raised the possibility of acute pancreatitis, whether biliary, toxic, metabolic, or drug-induced. Lupus enteritis with mesenteric vasculitis, peptic ulcer disease, and mesenteric ischemia in the setting of her known APS were also considered.

Lipase was 17 times the upper limit of normal. Hemoglobin was 10.2 g/dL, consistent with chronic inflammatory anemia. Liver enzymes, triglycerides, and calcium were normal (Table 1).

**Table 1:** Laboratory tests Hb : Hemoglobin; WBC: white blood count; PLT: platelets; CRP: C-reactive protein; AST: aspartate aminotransferase; ALT: alanine aminotransferase; GGT: gamma-glutamyl transferase; ALP: alkaline phosphatase; PT: prothrombin time

Lab test	Patient	Normal range
Hb (g/dl)	10.2	11.5-17.5
WBC (/mm3)	6730	3800-11000
PLT (/mm3)	290000	150000-445000
Lipase (UI/l)	1141	8-78
CRP (mg/l)	49	<8
AST (U/l)	26	5-34
ALT (U/l)	26	<55
GGT (U/l)	53	<55
ALP (U/l)	83	4-150
Total bilirubin (mg/l)	4	2-12
PT (%)	51.7	70-100
Calcium (mg/l)	92	85-101
Triglycerides (g/l)	1.32	<1.5

SLE-specific serology showed antinuclear antibodies (ANA) at 1:640, anti-double-stranded DNA (anti-dsDNA) antibodies at a titer of 1:20, anti-Sjögren's syndrome type A (anti-SSA), anti-Ro52, and anti-nucleosome antibodies, along with low complement levels (C3, C4) and proteinuria at 0.81 g/L on 24-hour collection. The Systemic Lupus Erythematosus Disease Activity Index (SLEDAI-2K) score was 20, indicating severe disease activity [6]. This validated composite tool evaluates 24 clinical and laboratory parameters across multiple organ systems [6]; in this patient, the score was driven by cutaneous vasculitis (8 points), arthritis (4 points), proteinuria (4 points), low complement (2 points), and anti-dsDNA positivity (2 points). Combined with migratory polyarthralgia and cutaneous vasculitis, this confirmed an active SLE flare.

**Figure 1:**
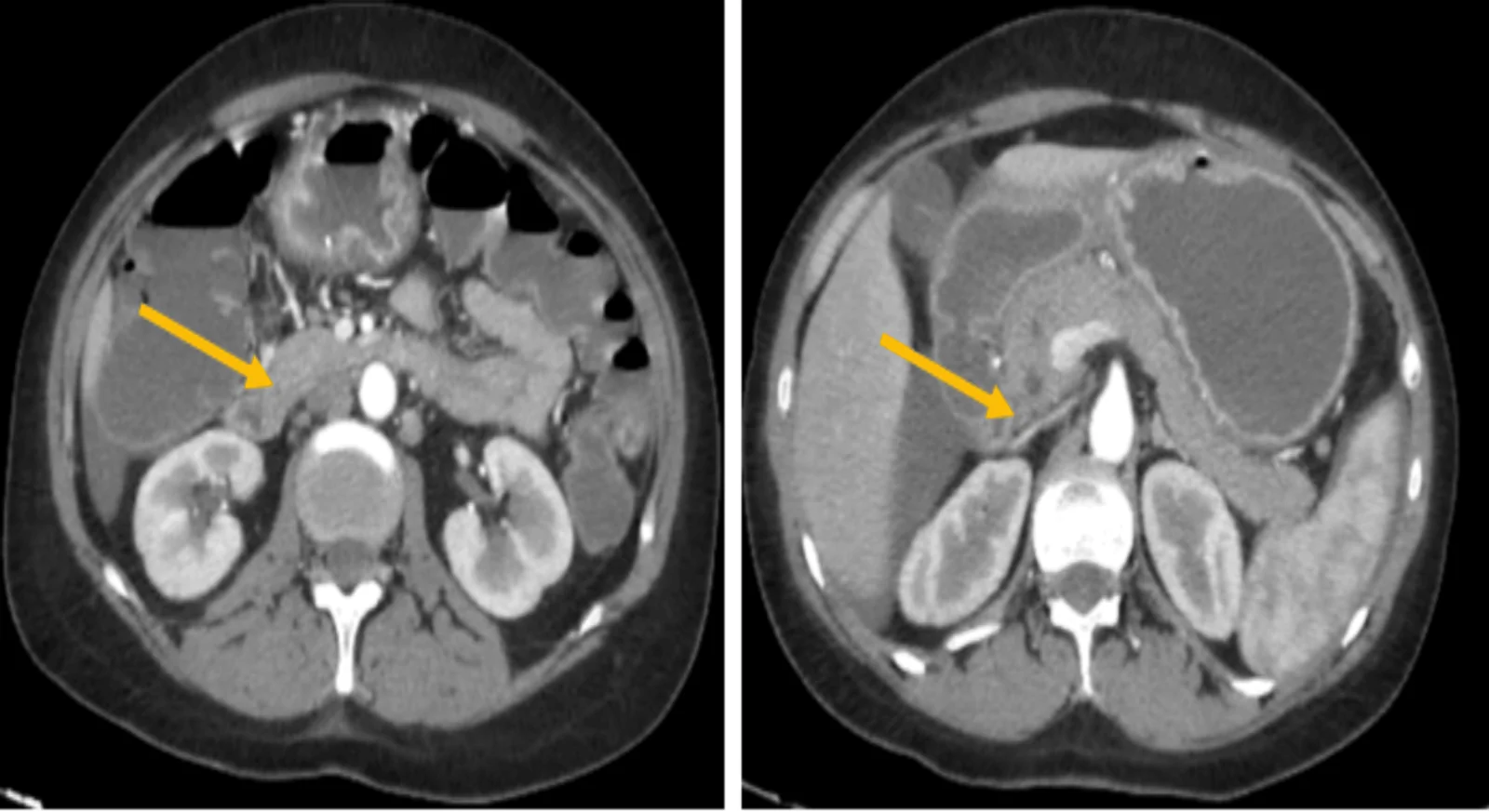
Abdominal CT showing acute pancreatitis (CTSI score, 2) Yellow arrow: pancreatic edema with peripancreatic fat stranding CTSI: CT Severity Index

Contrast-enhanced computed tomography (CT) (Figure 1) confirmed acute pancreatitis with a CT Severity Index (CTSI) score of 2 [7]. Abdominal ultrasound showed no gallstones or bile duct dilation. Small bowel wall thickening was noted incidentally.

The etiological workup included a thorough history, laboratory tests, and magnetic resonance cholangiopancreatography (MRCP), ruling out common causes: alcohol, toxic substances, medications, gallstones, hypertriglyceridemia, hypercalcemia, neoplasia, and biliopancreatic junction malformations.

The patient’s self-discontinuation of azathioprine prior to admission made drug-induced pancreatitis an unlikely cause. Given high SLE activity and no identifiable alternative etiology, we suspected lupus‑induced acute pancreatitis (LIAP).

The patient received three intravenous doses of 500 mg methylprednisolone during hospitalization. Pain resolved within 48 hours and she was discharged after seven days on corticosteroids and hydroxychloroquine. Her hospital course and subsequent follow-up were free of complications.

Follow-up at one month showed clinical improvement and a fall in CRP from 49 to 15 mg/L, which confirmed the response to immunosuppression and established the diagnosis of LIAP. Azathioprine was reintroduced at 100 mg daily, with prednisone 5 mg daily and hydroxychloroquine 200 mg twice daily. At her last consultation, 18 months after presentation, she had had no further episode of pancreatitis.

## Discussion

Acute pancreatitis occurs in approximately 0.7-1% of SLE patients, though reported rates reach up to 4% in some series [3,4]. Although gastrointestinal symptoms are common in SLE, pancreatitis remains relatively rare and often marks a severe disease course [5].

The underlying pathogenesis is multifactorial, involving immune complex deposition, small vessel vasculitis, complement activation, and microvascular thrombosis causing pancreatic ischemia [8,9].

Our patient had self-discontinued azathioprine and used hydroxychloroquine and corticosteroids inconsistently, which likely exacerbated systemic inflammation and contributed to pancreatic injury. Although azathioprine and corticosteroids have been implicated in drug-induced pancreatitis, she had stopped azathioprine before admission, making drug toxicity an unlikely cause [2,4]. A SLEDAI-2K of 20, with proteinuria and serological activity, is consistent with the SLE flare as the cause rather than drug toxicity.

Reaching this conclusion required excluding biliary, toxic, and metabolic causes before attributing the episode to SLE, in line with current acute pancreatitis guidelines [10]. It should be noted that the MRCP images are not available for inclusion, as the examination was performed at an external facility; this represents a limitation of the present report. ANA at 1:640, anti-dsDNA, anti-nucleosome antibodies, low complement, and a SLEDAI-2K of 20 all pointed to SLE as the cause.

Cases of acute pancreatitis preceding the diagnosis of SLE have also been reported [3,9].

Delayed diagnosis and organ failure are the main determinants of mortality [8].

A few adult cohorts have described SLE‑related acute pancreatitis, consistently linking it to high disease activity and reporting higher mortality rates. Table 2 summarizes the main series [11-14]. Across these series, SLE was active at the time of pancreatitis in nearly all cases. Severe forms occurred in 29-34% of patients, and mortality ranged from 16.6% (Muhammed et al.) to 40.7% (Bellamine et al.), reflecting differences in patient populations, disease severity, and treatment approaches [11,14].

**Table 2:** Main reported series of LIAP in adults IS: immunosuppressant; IV: intravenous; AP: acute pancreatitis; SLE: systemic lupus erythematosus; LIAP: lupus-induced acute pancreatitis

Study	Year	Country	Number of cases	Mean age (y)	Sex ratio F:M	Inaugural AP (%)	Active SLE at AP (%)	Severe AP (%)	LIAP treatment	Mortality (%)
Muhammed et al. [11]	2022	India	66	24	54:12	6	89.3	33.3	IV methylprednisolone or dexamethasone in 40 cases; IV cyclophosphamide in 24 cases	16.6
Yang et al. [12]	2012	China	27	30.1	25:2	0	100	29.6	Systemic glucocorticoids in most; some additional IS	37
Ben Dhaou et al. [13]	2013	Tunisia	6	36.3	5:1	66.7	100	33.3	High dose systemic corticosteroids	16.7
Bellamine et al. [14]	2023	Morocco	27	31	25:2	29.6	100	29.5	High-dose IV methylprednisolone pulses in 12 cases; IV Cyclophosphamide in 8 cases; azathioprine in 3 cases	40.7

High-dose corticosteroids are the primary treatment [2,4,5]. Our patient responded rapidly to methylprednisolone, consistent with outcomes reported across published series.

The main clinical difficulty is that corticosteroids are both the treatment and a potential cause. The indication must rest on a complete etiological workup before initiation, which is why establishing the diagnosis first matters.

Hydroxychloroquine was reintroduced at discharge for its protective effect on disease activity and prognosis [15].

Prognostic factors include necrotizing pancreatitis, infection, multiorgan involvement, and disease severity at presentation. Rim et al. showed that SLE patients hospitalized with acute pancreatitis had longer hospital stays and higher mortality compared to non-SLE controls [8]. Early diagnosis, absence of necrosis, and early immunosuppression are associated with favorable outcomes [10,11].

Complement levels (C3, C4) and anti-dsDNA titers may help predict disease activity and pancreatic involvement [16].

In refractory or fulminant cases, plasma exchange has been reported, though evidence remains limited to small series [17].

Our patient has now been followed for 18 months without recurrence, on azathioprine 100 mg daily throughout. Tolerance of the drug she had been taking before the episode argues against drug-induced pancreatitis, though longer follow-up would be needed to confirm sustained remission.

With fewer than 150 cases across the four main published series, treatment recommendations rely on expert opinion rather than controlled evidence.

## Conclusions

In SLE patients with acute abdominal pain during a flare, acute pancreatitis should be considered early. In this patient, self-discontinuation of azathioprine and a SLEDAI-2K of 20 pointed to disease activity rather than drug toxicity as the cause, a distinction that required a systematic etiological workup before starting corticosteroids. Early corticosteroid initiation was associated with rapid improvement, and the patient remained free of recurrence at 18 months on azathioprine.

As with all single case reports, the conclusions are limited in their generalizability, and prospective multicenter studies are needed to validate the diagnostic and therapeutic approach described.
